# Parâmetros de *Strain* Diastólico Estão Associados à Mortalidade em Curto Prazo e à Reinternação em Pacientes com Insuficiência Cardíaca Avançada

**DOI:** 10.36660/abc.20230670

**Published:** 2024-08-14

**Authors:** Sefa Tatar, Abdullah İcli, Alpay Arıbaş, Nazire Belgin Akilli, Hakan Akilli, Ahmet Lütfi Sertdemir

**Affiliations:** 1 Necmettin Erbakan Universitesi Konya Turquia Necmettin Erbakan Universitesi – Kardiyoloji, Konya – Turquia; 2 Konya City Hospital Konya Turquia Konya City Hospital, Konya – Turquia

**Keywords:** Insuficiência Cardíaca, Readmissão do Paciente, Mortalidade

## Abstract

**Fundamento:**

A insuficiência cardíaca é uma das principais causas de hospitalização e mortalidade em todo o mundo e representa um grande fardo económico para os sistemas de saúde. A identificação de fatores prognósticos em pacientes com IC é de grande importância para estabelecer estratégias de manejo ideais e evitar procedimentos invasivos e dispendiosos desnecessários em pacientes em estágio terminal.

**Objetivos:**

No presente estudo, nosso objetivo foi investigar a associação entre parâmetros de *strain* diastólico, incluindo E/e’ SR, e resultados de curto prazo em pacientes com IC avançada.

**Métodos:**

O estudo populacional incluiu 116 pacientes com insuficiência cardíaca avançada com fração de ejeção reduzida (ICFEr) avançada. Avaliações clínicas, laboratoriais e ecocardiográficas dos pacientes foram realizadas nas primeiras 24 horas de internação. Os pacientes foram acompanhados por um mês e qualquer reinternação por piora dos sintomas de IC e qualquer mortalidade foi registrada. O nível de significância adotado na análise estatística foi de 5%.

**Resultados:**

A E/e’ SR foi significativamente maior no grupo de pacientes em comparação ao grupo controle (p=0,001). Durante o acompanhamento de um mês, 13,8% dos pacientes morreram e 37,1% dos pacientes foram reinternados. NT-ProBNP sérico (p=0,034) e E/e’ SR (p=0,033) foram considerados preditores independentes de mortalidade e o uso de IECA (p=0,027) e *strain* 3C apical (p=0,011) foram considerados independentes preditores de reinternação no grupo de pacientes.

**Conclusão:**

Os resultados do presente estudo prospectivo demonstram que a E/e’ SR medida pela ecocardiografia com *speckle tracking* é um preditor independente e sensível de mortalidade em curto prazo em pacientes com ICFEr avançada e pode ter um papel na identificação de pacientes com ICFEr em estágio terminal.

**Figura Central:**
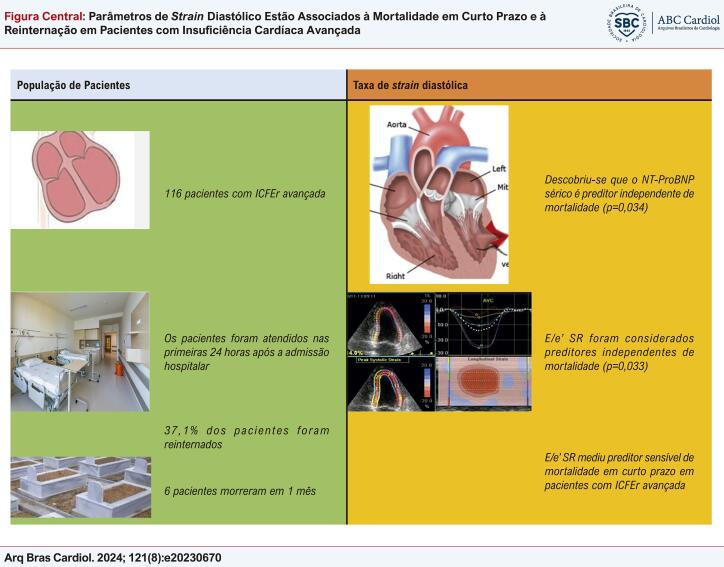
: Parâmetros de *Strain* Diastólico Estão Associados à Mortalidade em Curto Prazo e à Reinternação em Pacientes com Insuficiência Cardíaca Avançada Ecocardiografia de strain diastólico e mortalidade em curto prazo em pacientes com ICFEr avançada. ICFER: insuficiência cardíaca com fração de ejeção reduzida; SR: taxa de strain; NT-ProBNP: O pró-hormônio N-terminal do peptídeo natriurético cerebral.

## Introdução

A insuficiência cardíaca (IC) é uma das principais causas de complicações fatais e não fatais e continua a ser um problema de saúde crescente em todo o mundo.^1^ A prevalência de IC na população adulta é relatada em cerca de 1% a 2%, mas a taxa é mais de 10% em indivíduos mais velhos com mais de 70 anos de idade.^2 , 3^ Apesar de todas as novas opções terapêuticas, a IC ainda está associada a uma elevada taxa de mortalidade e o tratamento da IC representa um grande fardo económico para os sistemas de saúde.^4^ A avaliação precisa do estado clínico e do prognóstico em pacientes com IC é de suma importância para estabelecer um tratamento adequado, estratégias e evitar procedimentos invasivos e dispendiosos desnecessários em pacientes em fase terminal.

Além da avaliação clínica e dos exames bioquímicos, a avaliação ecocardiográfica é parte indispensável da avaliação da IC. As funções sistólica e diastólica do ventrículo esquerdo são preditores bem definidos para o resultado cardiovascular em pacientes com IC.^5 , 6^ A ecocardiografia com *speckle tracking* (STE) é um novo método para avaliar a função ventricular esquerda por meio da quantificação da deformação miocárdica ( *strain* ) e da taxa de deformação (taxa de *strain* ).^7^ A relação entre a velocidade de enchimento transmitral precoce e o taxa de *strain* diastólica precoce (E/e’ SR) medida por STE também emergiu como um marcador confiável da pressão de enchimento ventricular esquerdo e um preditor sensível de desfechos cardiovasculares em pacientes com IC crônica.^8 , 9^ Por outro lado, há dados limitados sobre a associação entre parâmetros de STE incluindo E/e’ SR e prognóstico na IC avançada com sintomas graves (estado funcional Classe III-IV da New York Heart Association), disfunção cardíaca grave e congestão pulmonar ou sistêmica que requer diuréticos intravenosos.^10^

No presente estudo, nosso objetivo foi investigar a associação entre parâmetros de *strain* diastólico, incluindo E/e’ SR medido por STE, e resultados de curto prazo em pacientes com IC avançada.

## Métodos

### População do estudo

No presente estudo, foram incluídos 176 pacientes com IC avançada com fração de ejeção reduzida (ICFEr) (FE ≤40%, estado funcional Classe III-IV da New York Heart Association, congestão pulmonar ou sistêmica que requer diuréticos intravenosos, mais de duas hospitalizações ou visitas recorrentes ao serviço de emergência no último ano, deterioração progressiva da função renal, desenvolvimento de caquexia sem causa identificável, incapacidade de usar inibidores da ECA devido a insuficiência renal ou hipotensão, agravamento da IC ou intolerância a betabloqueadores devido à hipotensão, desenvolvimento de resistência aos diuréticos e aumento da dose de furosemida acima de ≥160 mg/dL, bem como desenvolvimento de hiponatremia) e 58 indivíduos sem doença cardíaca conhecida.^10^ Não houve critério definindo o tamanho da amostra utilizada na pesquisa, sendo estipulado por conveniência. Não tivemos um critério que definisse o tamanho da amostra para cada um dos dois grupos examinados. Quarenta e cinco pacientes com ICFEr avançada foram excluídos do estudo. Entre os pacientes excluídos, 10 apresentavam insuficiência aórtica avançada, 5 apresentavam insuficiência mitral avançada, 3 apresentavam estenose aórtica avançada e 7 apresentavam estenose mitral avançada. Além disso, 10 pacientes foram excluídos do estudo por doença protética valvar aórtica e 5 por doença protética valvar mitral. Além disso, 5 pacientes foram excluídos do estudo por terem cardiodesfibrilador implantável (CDI) instalado e necessidade de suporte inotrópico.

Um total de 131 pacientes foram encaminhados para avaliação ecocardiográfica. Todos os pacientes foram submetidos à avaliação ecocardiográfica após avaliação inicial no pronto-socorro/ambulatório antes da admissão na unidade de terapia intensiva (UTI)/enfermaria e antes da terapia diurética. Nesta fase, outros 15 pacientes foram excluídos do estudo devido à má qualidade da imagem ecocardiográfica. A população final do estudo incluiu 116 pacientes com ICFEr avançada como “grupo de pacientes” e 58 indivíduos saudáveis como “grupo controle”. Dos 116 pacientes, 40 foram internados na UTI e 76 na enfermaria de cardiologia após avaliação inicial. Dos pacientes internados em enfermaria, 3 necessitaram de transferência para UTI durante a internação.

**Figura 1:**
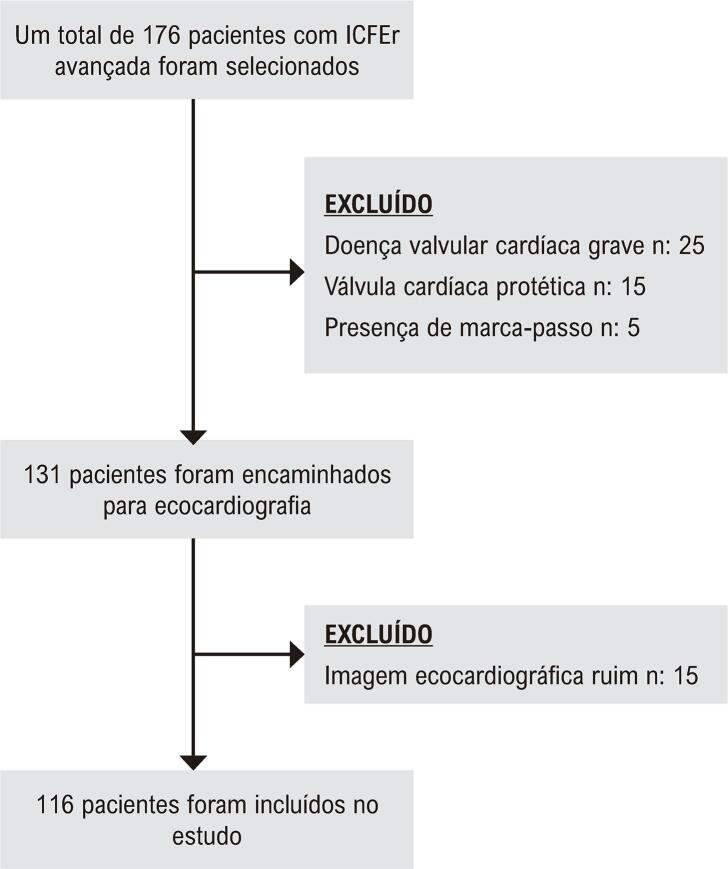
– *Fluxograma do paciente. 176 pacientes com ICFEr avançada foram incluídos no estudo. 45 pacientes foram excluídos do estudo devido à presença de doença valvar cardíaca grave, prótese valvar cardíaca e história de marca-passo. 15 pacientes foram excluídos devido à má qualidade da imagem ecocardiográfica. A população final do estudo incluiu 116 pacientes com ICFEr avançada. ICFEr: insuficiência cardíaca avançada com fração de ejeção reduzida.*

O fluxograma do estudo é apresentado na Figura 1 . O nível de peptídeo natriurético pró-cérebro N-terminal (ProBNP) foi medido em todos os pacientes, além de exames de sangue de rotina. Os pacientes foram acompanhados por um mês e qualquer reinternação por piora dos sintomas de IC e qualquer mortalidade foi registrada. Os desfechos desfavoráveis dos pacientes foram avaliados tanto por telefone quanto por meio de prontuários. O protocolo do estudo foi aprovado pelo comitê de ética local (2019/1724) e o consentimento informado foi obtido de todos os pacientes.

### Ecocardiografia

**Figura 2:**
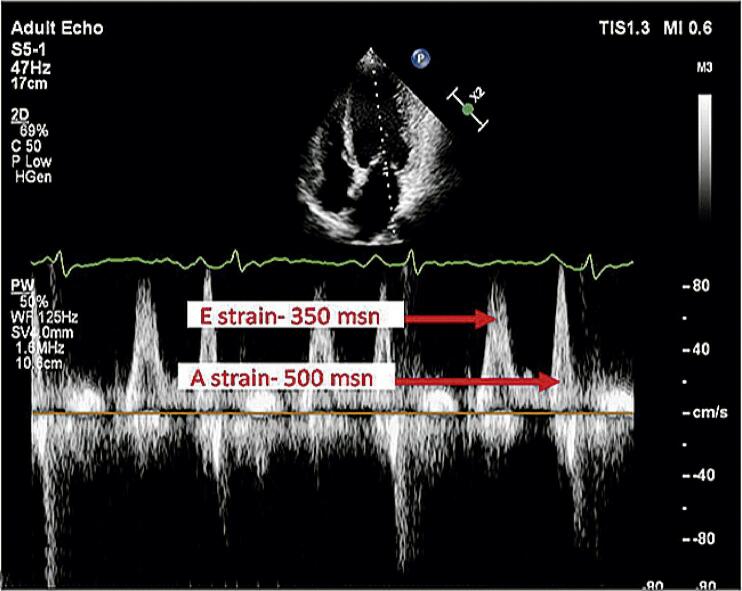
– *A relação entre a velocidade de strain e a atividade elétrica do coração na ecocardiografia. Os tempos de medição elétrica foram medidos desde o ponto de pico da onda QRS até os pontos de pico da onda E e da onda A no solo horizontal.*

**Figura 3:**
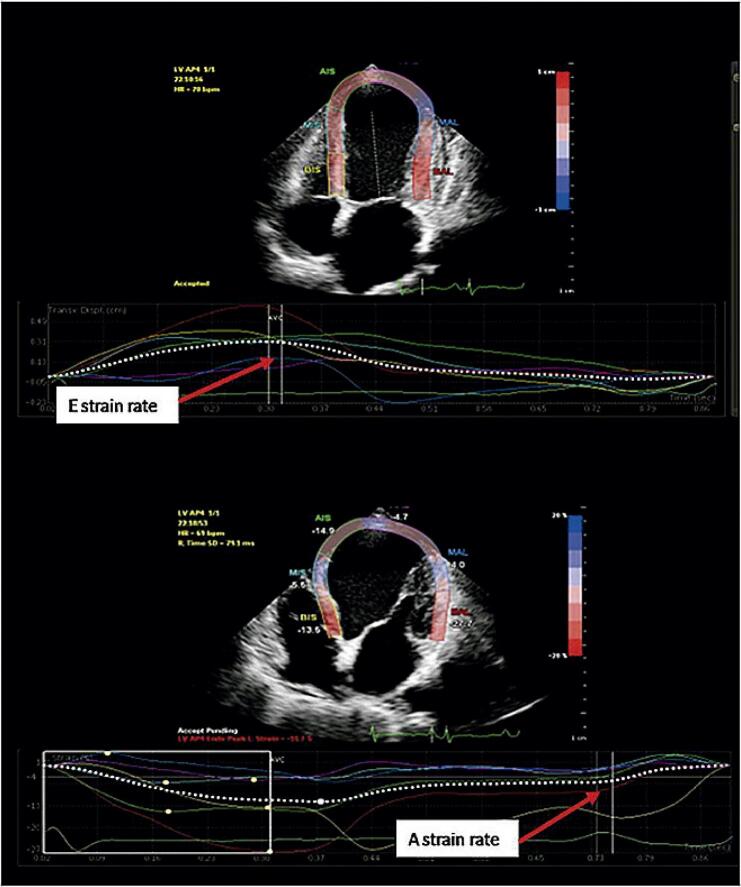
– *Taxa de strain E (1/s) e taxa de strain A (1/s) da janela apical.*

A avaliação ecocardiográfica transtorácica foi realizada com aparelho de ultrassom Philips Epiq 7C (Bothell, WA, EUA) com transdutor de 5-1 MHz. As medidas ecocardiográficas foram obtidas por dois cardiologistas experientes, utilizando técnicas padrão e imagens sugeridas pelas diretrizes da *American Echocardiography Association* .^11^ A fração de ejeção do ventrículo esquerdo (FEVE) foi calculada pelo método biplano de Simpson. Foram obtidas visualizações paraesternal eixo longo, paraesternal eixo curto, apical 4 câmaras e apical eixo longo. Todas as imagens foram maiores que 60 fps e obtidas durante pelo menos 5 ciclos cardíacos. Foram calculados os valores do *strain* longitudinal global (SLG) para todas as paredes do ventrículo esquerdo. Tanto os pontos basais quanto o ápice do miocárdio foram determinados em cada janela nas imagens SR. Foram calculadas a distância entre o pico R do complexo QRS e o ponto de pico da frequência E mitral e a distância entre o pico R do complexo QRS e o ponto de pico da frequência A mitral ( Figura 2 ). Os valores de e’ SR foram calculados a partir dos períodos obtidos ( Figura 3 ). O influxo mitral foi medido na onda diastólica inicial (E) e na onda diastólica tardia (A) em 5 a 10 ciclos cardíacos do lado distal de 1 cm das extremidades da valva mitral na visão apical de quatro câmaras através do Doppler de onda pulsada (PW), e sua média foi calculado. O valor E mitral foi então dividido em frequência e’ SR e o valor absoluto de E/e’ SR foi calculado. Se a variabilidade da RS medida por dois operadores fosse superior a 5%, o paciente era excluído. Se a diferença entre duas medidas for inferior a 5%, a média aritmética dos dois valores foi calculada e utilizada para as análises.

### Análise estatística

O software SPSS ( *Statistical Package for Social Sciences* ) Windows 22.0 foi utilizado para análises estatísticas. A distribuição normal das variáveis contínuas foi avaliada pelo teste de Kolmogorov-Smirnov. Os dados com distribuição normal foram expressos como média±desvio padrão, enquanto os dados sem distribuição normal foram expressos como mediana [intervalo interquartil (IQR)]. As variáveis categóricas foram expressas como frequências absolutas (n) e relativas (%) e a associação entre variáveis categóricas foi avaliada pelo teste Qui-quadrado. O teste-t de Student independente foi utilizado para comparar parâmetros normalmente distribuídos. Parâmetros distorcidos foram comparados usando o teste U de Mann-Whitney. Análises de regressão logística foram utilizadas para avaliar o valor prognóstico dos parâmetros do SLG para prever reinternação e mortalidade. Os valores de corte ideais foram determinados pela análise dos valores de sensibilidade e especificidade derivados da análise da curva característica de operação do receptor (ROC). Qualquer valor de p abaixo de 0,05 (p<0,05) foi aceito como estatisticamente significativo.

## Resultados

As características demográficas, clínicas, ecocardiográficas e laboratoriais dos grupos de pacientes e controle são apresentadas na Tabela 1 . Os níveis séricos de creatinina e ureia foram significativamente maiores no grupo de pacientes (p=0,018 e p=0,001, respectivamente). Além disso, houve diferenças significativas entre os dois grupos em relação aos parâmetros ecocardiográficos, incluindo parâmetros de strain diastólico ( Tabela 1 ). A E/e’ SR foi significativamente maior no grupo de pacientes em comparação ao grupo controle [188,8(323,0) vs 54,1(21,0), p=0,001].

**Tabela 1:** – Características demográficas, clínicas e laboratoriais dos grupos de pacientes e controle

	Grupo de Pacientes (n=116) Média±DP/Mediana (IQR)	Grupo de controle (n=58) Média±DP/Mediana (IQR)	p
**Parâmetros**			
**Anos de idade**	67,7±12,8	68,1±11,2	0,810
**Gênero**			0,524
Masculino, n (%)	71 (61,2%)	36 (62,1%)	
Feminino, n (%)	45 (38,8%)	22 (37,9%)	
**DAC, n (%)**			**0,019**
Nenhum	41 (35,3%)	35 (60,3%)	
Médica	15 (12,9%)	5 (8,6%)	
ACTP	39 (33,6%)	11 (19%)	
CRM	21 (18,1%)	7 (12,1%)	
**Hipertensão, n (%)**	84 (72,4%)	47 (81%)	0,145
**Fumante, n (%)**	40 (34,5%)	15 (25,9%)	0,321
**Diabetes mellitus, n (%)**	52 (44,8%)	34 (58,6%)	0,06
**Hiperlipidemia, n (%)**	49 (42,2%)	23 (39,7%)	0,43
**Acidente vascular cerebral, n (%)**	16 (13,8%)	6 (10,3%)	0,35
**DRC, n (%)**	53 (45,7%)	21 (36,2%)	0,151
**Hemoglobina (g/dl)**	12,4±2,2	12,9±2,2	0,828
**Hematócrito**	38,4±6,7	39,8±6,4	0,364
**VCM (fL)**	83,7±7,5	86,5±6,6	0,275
**Contagem de plaquetas (x109/L)**	246,5±96,3	252,0±78,3	0,705
**Sódio (mmol/L)**	138,0±3,7	138,8±3,4	0,837
**Potássio (mmol/L)**	4,6±0,7	4,3±0,6	0,182
**TFG (ml/min)**	51(29)	72(40)	**0,002**
**Ureia (mg/dl)**	57(39)	40(29)	**0,001**
**Creatina (mg/dl)**	1,39(1)	0,98(1)	**0,018**
**AST (U/L)**	18(12)	17(13)	0,870
**ALT (U/L)**	15(14)	17(18)	0,908
**Contagem de leucócitos (x109/L)**	9,1 (3,9)	8,3(4,5)	0,308
**Ecocardiografia**			
**Diâmetro diastólico final (mm)**	56,8±7,7	45,3±4,5	**0,001**
**Diâmetro sistólico final (mm)**	44,3±8,7	27,8±4,0	**0,001**
**Átrio esquerdo (mm)**	43,5±7,0	34,8±6,4	**0,001**
**Pressão arterial pulmonar (mmHg)**	35(21)	30(9)	**0,001**
**Volume diastólico final (ml)**	182,8(90)	42,4(32)	**0,001**
**Volume sistólico final (ml)**	122,5(60)	17,3(14)	**0,001**
**Fração de ejeção (%)**	30,6(11)	57,1(13)	**0,001**
***Strain* apical 3C (%)**	-6,7±2,9	-9,3 ±3,2	**0,001**
***Strain* longitudinal global (%)**	-7,2±2,46	-10,5±2,6	**0,001**
**E SR, 1/s**	0,34(0,4)	1,0(0,5)	**0,001**
**a SR, 1/s**	0,3(0)	1.1(1)	**0,001**
**E/e'SR**	188,8(323,0)	54,1(21,0)	**0,001**

*ALT: alanina aminotransferase; AST: aspartato aminotransferase; CRM: cirurgia de revascularização do miocárdio; DAC: doença arterial coronariana; DRC: doença renal crônica; TFG: taxa de filtração glomerular; VCM: volume corpuscular médio; ACTP: angioplastia coronária transluminal percutânea; SR: taxa de strain. Valores em negrito indicam valores de p significativos.*

### Preditores de mortalidade no grupo de pacientes

Durante um mês de acompanhamento, 16 (13,8%) pacientes morreram. As características demográficas, clínicas, ecocardiográficas e laboratoriais dos grupos de mortalidade e sobrevida são apresentadas na Tabela 2 . Betabloqueador e o uso de inibidores da enzima conversora de angiotensina (IECA) foram menos comuns no grupo de mortalidade. Por outro lado, o nível sérico de proBNP foi significativamente maior no grupo de mortalidade. Além disso, a E/e’ SR também foi significativamente maior no grupo de pacientes em comparação ao grupo controle. Os diâmetros diastólico final do ventrículo esquerdo e do átrio esquerdo foram significativamente menores no grupo de mortalidade

**Tabela 2:** – Características demográficas, laboratoriais e ecocardiográficas dos grupos mortalidade e sobrevivência

	Grupo de Mortalidade (n=16) Média±DP/Mediana (IQR)	Grupo de Sobrevivência (n=100) Média±DP/Mediana (IQR)	p
**Anos de idade**	69,1±12,4	67,5±12,9	0,644
**Gênero**			0,236
Masculino, n (%)	8 (50,0%)	63 (63,0%)	
Feminino, n (%)	8 (50,0%)	37 (37,0%)	
**Tempo total de internação (dias)**	7 (4-9)	7 (3-9)	0,690
**Hipertensão, n (%)**	10 (62,5%)	74 (74%)	0,251
**Diabetes mellitus, n (%)**	7 (43,8%)	45 (45%)	0,573
**Hiperlipidemia, n (%)**	5 (31,2%)	44 (44%)	0,249
**Betabloqueador, n (%)**	8 (50%)	75 (75%)	**0,043**
**Diurético, n (%)**	8 (50%)	69 (69%)	0,115
**IECA, n (%)**	5 (31,2%)	59 (59%)	**0,036**
**ARM, n (%)**	3 (18,8%)	34 (34%)	0,178
**Diâmetro diastólico final (mm)**	52,7±8,7	57,4±7,3	**0,021**
**Diâmetro sistólico final (mm)**	42,6±9.1	44,6±8.6	0,384
**Diâmetro do átrio esquerdo (mm)**	40,4±8,2	44,0±6,6	**0,049**
**TFG (ml/min)**	41 (34)	51 (20)	0,216
**ProBNP (ng/L)**	13800 (30747)	4860 (9174)	**0,002**
**Volume diastólico final (ml)**	166,6 (138)	182,3 (76)	0,876
**Volume sistólico final (ml)**	116,4 (97)	121,2 (51)	0,719
**Fração de ejeção (%)**	31,7 (10)	30,3 (11)	0,513
***Strain* apical 4C (%)**	-7,5±2,4	-7,3±2,7	0,777
***Strain* apical 3C (%)**	-6,2±2,3	-6,7 ±3,0	0,623
***Strain* apical 2C (%)**	-7,4±4,2	-6,7±2,8	0,503
***Strain* longitudinal global (%)**	-7,4±2,1	-7,2±2,5	0,742
**E SR, 1/s**	0,1 (0,3)	0,36 (0,3)	**0,004**
**A SR, 1/s**	0,3 (0,0)	0,3 (0,0)	0,774
**E/e'SR**	600,0 (679,0)	184,4 (318,0)	**0,009**

*IECA: inibidor da enzima conversora de angiotensina; BNP: peptídeo natriurético cerebral; TFG: taxa de filtração glomerular; ARM: antagonista do receptor mineralocorticoide; SR: taxa de strain. Valores em negrito indicam valores de p significativos.*

Análises de regressão logística univariada e multivariada foram realizadas para identificar preditores de mortalidade no grupo de pacientes e os resultados dessas análises são apresentados na Tabela 3 . A análise de regressão multivariada revelou que o nível sérico de proBNP e E/e’ SR foram preditores independentes de mortalidade no grupo de pacientes durante o acompanhamento de um mês.

**Tabela 3:** – Análises de regressão univariada e multivariada para identificação de preditores de mortalidade no grupo de pacientes

Parâmetros	Análise univariada		p	Análise multivariada		p
	OR	IC 95%		OR	IC 95%	
Betabloqueador*, n (%)	3,0	1.019-8.829	**0,046**	2.753	0,824-9,203	0,100
Diurético*, n (%)	2.226	0,765-6,474	0,142	0,861	0,157-4,714	0,863
IECA*, n (%)	3.166	1.023-9.798	**0,046**	1.356	0,306-5,998	0,688
**ARM, n (%)**	2.232	0,595-8,372	0,234	-	-	-
Diâmetro diastólico final* (mm)	0,916	0,850-0,988	**0,024**	0,956	0,866-1,055	0,369
**Diâmetro sistólico final (mm)**	0,972	0,913-1,036	0,381	-	-	-
**FEVE (%)**	0,983	0,927-1,043	0,569	-	-	-
Diâmetro do átrio esquerdo* (mm)	0,927	0,858-1,001	**0,053**	0,925	0,851-1,004	0,064
ProBNP* (ng/L)	1.000	1.000-1.000	**0,029**	1.000	1.000-1.000	**0,034**
E/e'SR*	1.001	1.000-1.001	**0,048**	1.001	1.000-1.001	**0,033**

IECA: inibidor da enzima conversora de angiotensina; BNP: peptídeo natriurético cerebral; IC: intervalo de confiança; FEVE: fração de ejeção do ventrículo esquerdo; ARM: antagonista do receptor mineralocorticoide; OR: razão de chances; SR: taxa de strain. *Esses parâmetros são incluídos na análise multivariada. Valores em negrito indicam valores de p significativos.

A análise das características operacionais do receptor demonstrou que um valor de corte de 218,75 para E/e’ SR teve sensibilidade e especificidade de 86,7 e 58,0% para predizer mortalidade no grupo de pacientes. Um valor de corte de 6.326,50 ng/L para o nível sérico de proBNP teve sensibilidade e especificidade de 85,7 e 56,2% para prever mortalidade.

### Preditores de reinternação no grupo de pacientes

Durante um mês de acompanhamento, 43 (37,1%) pacientes foram reinternados devido ao agravamento dos sintomas de IC. As características demográficas, clínicas, ecocardiográficas e laboratoriais dos grupos de reinternação e não reinternação são mostradas na Tabela 4 . O uso de IECA foi mais comum no grupo de reinternação. Além disso, a deformação apical 3C foi significativamente pior no grupo de hospitalização em comparação com o grupo de não reinternação. Por outro lado, os níveis séricos de proBNP e E/e’ SR foram semelhantes entre os grupos.

**Tabela 4:** – Características demográficas, laboratoriais e ecocardiográficas dos grupos reinternação e não reinternação

	Grupo de Reinternação (n=43) Média±DP/Mediana (IQR)	Grupo de não reinternação (n=73) Média±DP/Mediana (IQR)	p
**Anos de idade**	66,5±11.9	68,4±13,3	0,452
**Gênero**			0,845
Masculino, n (%)	27 (62,8%)	44 (60,3%)	
Feminino, n (%)	16 (37,2%)	29 (39,7%)	
**Tempo total de internação (dias)**	6 (3-9)	7 (3-9)	0,662
**Hipertensão, n (%)**	34 (79,1%)	50 (68,5%)	0,155
**Diabetes mellitus, n (%)**	24 (55,8%)	28 (38,4%)	0,551
**Hiperlipidemia, n (%)**	19 (44,2%)	30 (41,1%)	0,447
**Betabloqueador, n (%)**	33 (76,7%)	50 (68,5%)	0,231
**Diurético, n (%)**	32 (74,4%)	45 (61,6%)	0,114
**IECA, n (%)**	29 (67,4%)	35 (47,9%)	**0,032**
**ARM, n (%)**	17 (39,5%)	20 (27,4%)	0,126
**Diâmetro diastólico final (mm)**	57,1±7,1	56,5±8,0	0,686
**Diâmetro sistólico final (mm)**	44,1±8.7	44,5±8.7	0,824
**Diâmetro do átrio esquerdo (mm)**	44,4±7,1	43,0±6,6	0,292
**TFG (ml/min)**	47,0 (17,0)	51,5 (34,0)	0,224
**ProBNP (ng/L)**	5560 (8721)	7368 (15593)	0,554
**Volume diastólico final (ml)**	196,8 (92)	169,8 (78)	0,154
**Volume sistólico final (ml)**	129,9 (64)	117 (48)	0,117
**Fração de ejeção (%)**	33 (11)	30 (11)	0,268
***Strain* apical 4C (%)**	-6,7±2,4	-7,63±2,7	0,116
***Strain* apical 3C (%)**	-5,57±2,7	-6,7 ±3,0	**0,010**
***Strain* apical 2C (%)**	-6,4±2,9	-6,9±2,9	0,457
***Strain* longitudinal global (%)**	-7,0±2,6	-7,4±2,36	0,462
**E SR, 1/s**	0,37 (0,3)	0,32 (0,4)	0,817
**A SR, 1/s**	0,32 (0)	0,3 (0)	0,765
**E/e'SR**	178,3 (305)	221,8 (473)	0,470

*IECA: inibidor da enzima conversora de angiotensina; BNP: peptídeo natriurético cerebral; TFG: taxa de filtração glomerular; ARM: antagonista do receptor mineralocorticoide; SR: taxa de strain. Valores em negrito indicam valores de p significativos.*

Análises de regressão univariada e multivariada foram realizadas para identificar preditores de reinternação no grupo de pacientes e os resultados dessas análises são apresentados na Tabela 5 . A análise de regressão multivariada revelou que o uso de IECA e o *strain* apical 3C foram preditores independentes de reinternação no grupo de pacientes durante um acompanhamento de -mês.

**Tabela 5:** – Análises de regressão univariada e multivariada para identificação de preditores de reinternação no grupo de pacientes

Parâmetros	Análise univariada		p	Análise multivariada		p
	OR	IC 95%		OR	IC 95%	
**Hipertensão, n (%)**	0,575	0,237-1,395	0,221	-	-	-
Diurético*, n (%)	0,552	0,240-1,269	0,162	0,756	0,269-2,218	0,597
IECA*, n (%)	0,445	0,203-0,976	**0,043**	2.525	1.111-5.738	**0,027**
ARM*, n (%)	0,577	0,260-1,283	0,177	0,832	0,317-2,186	0,709
**Volume diastólico final (ml)**	1.003	0,997-1,009	0,264	-	-	-
**Volume sistólico final (ml)**	1.005	0,997-1,012	0,247	-	-	-
**FEVE (%)**	0,969	0,923-1,016	0,192	-	-	-
Strain apical 3C* (%)	1.138	1.023-1.265	**0,017**	1.154	1.033-1.288	**0,011**
Strain apical 4C* (%)	1.119	1.008-1.241	**0,035**	1.039	0,898-1,202	0,609

IECA: inibidor da enzima conversora de angiotensina; IC: intervalo de confiança; FEVE: fração de ejeção do ventrículo esquerdo; ARM: antagonista do receptor mineralocorticoide; OR: razão de chances. *Esses parâmetros estão incluídos na análise multivariada. Valores em negrito indicam valores de p significativos.

A análise das características operacionais do receptor demonstrou que um valor de corte de -5,55% para o *strain* apical 3C teve sensibilidade e especificidade de 76,7 e 50,7% para prever reinternação no grupo de pacientes. A Figura Central resume as principais informações do manuscrito.

## Discussão

No presente estudo prospectivo, revelamos que a E/e’ SR medida pelo STE e o nível sérico de proBNP foram preditores independentes de mortalidade em pacientes com ICFEr avançada durante o acompanhamento de um mês. Por outro lado, o *strain* apical 3C foi o único preditor de reinternação no mesmo grupo de pacientes. Existem outros estudos na literatura que investigam o papel prognóstico dos parâmetros de STE, incluindo E/e’ SR em pacientes com IC, mas nosso estudo é único para sua população de pacientes (pacientes com ICFEr avançada) e tempo de avaliação ecocardiográfica (dentro das primeiras 24 horas após hospitalização). admissão).^9^

Em pacientes com IC, o relaxamento miocárdico é prejudicado e o enchimento ventricular esquerdo diminui. Como resultado, ocorre um aumento da pressão diastólica e os pacientes começam a desenvolver sintomas de IC. Com o aumento do tônus venoso, o desenvolvimento de retenção de sódio e a ativação de vias neuro-hormonais, a pressão diastólica do ventrículo esquerdo aumenta ainda mais, a rigidez ventricular esquerda aumenta significativamente e ocorre edema pulmonar nos pacientes. Embora a contribuição da pressão atrial tente aumentar o débito cardíaco para compensar esta situação, o enchimento diastólico é limitado devido a uma resposta ventricular severa. Essa situação faz com que a dispneia aos esforços e a congestão pulmonar piorem nos pacientes. Especialmente a dilatação e disfunção atrial esquerda devem ser observadas como um guia importante em pacientes com IC diastólica.^12 , 13^ Embora tenham sido tentadas explicações fisiopatológicas para serem explicadas desta forma, a fisiopatologia ainda não foi claramente explicada.

A imagem de *strain* miocárdico com STE fornece importantes informações diagnósticas e prognósticas adicionais sobre a ecocardiografia básica e a imagem Doppler tecidual em pacientes com IC.^14^ Estudos anteriores demonstraram que o SLG foi um preditor independente de mortalidade por todas as causas em pacientes com ICFEr e adicionou valor prognóstico incremental significativo para os fatores de risco bem conhecidos, como a FEVE.^15 , 16^ Por outro lado, no presente estudo, não detectamos associação entre SLG e mortalidade/rehospitalização em pacientes com ICFEr avançada. A diferença mais importante entre esses estudos e os nossos é que investigamos uma amostra de pacientes com ICFEr avançada, mas outros estudos tiveram uma população global com IC. Essa discrepância também pode ser resultado da população relativamente pequena de pacientes do presente estudo.

Em sua grande série avaliando características clínicas e desfechos de pacientes com IC avançada, Javaloyes et al. relataram que a maioria dos pacientes tinha um fenótipo “quente e úmido”.^17^ A taxa de mortalidade em 1 ano foi de 30,8% no grupo de estudo e mais alta nos pacientes com fenótipo “frio e seco”. Como resultado, concluíram que a hipoperfusão estava relacionada a um aumento da taxa de mortalidade intra-hospitalar e em 1 ano em pacientes com IC avançada, em linha com estudos anteriores.^18^ Por outro lado, em pacientes sem hipoperfusão, os preditores de mortalidade são não está claro. No presente estudo revelamos que a avaliação ecocardiográfica precoce e a avaliação não invasiva da pressão de enchimento do ventrículo esquerdo através da relação entre a velocidade de enchimento precoce transmitral e a taxa de *strain* diastólica precoce (E/e’ SR), além de parâmetros clínicos e bioquímicos, pode fornecer informações valiosas sobre o prognóstico de pacientes com IC avançada com fenótipo “quente e úmido”.

A relação entre a velocidade de enchimento transmitral precoce e a taxa de *strain* diastólica precoce (E/e’ SR) obtida por STE emergiu como uma medida confiável das pressões de enchimento ventricular esquerdo que contorna as limitações técnicas dos parâmetros derivados do Doppler.^19 , 20^ E/e ‘SR é um parâmetro menos dependente da carga do que E/e’ e não é afetado significativamente pela sobrecarga de volume, o que o torna um marcador útil da pressão de enchimento do ventrículo esquerdo em pacientes com ICFEr. Estudos recentes mostraram que a RS de E/e’ foi um forte preditor de mortalidade e piores resultados em diversas condições, incluindo ICFER, fibrilação atrial, estenose aórtica e diabetes tipo 2.^9 , 21 - 24^ Em consonância com esses achados, revelamos que E/e’ SR foi um preditor independente de mortalidade em curto prazo em pacientes com ICFEr avançada, juntamente com o nível sérico de proBNP. Tanto o E/e’ SR quanto os níveis séricos de proBNP apresentaram alta sensibilidade, mas baixa especificidade para a predição de mortalidade em nosso grupo de pacientes.

Apesar das enormes melhorias nas estratégias de gestão de pacientes com ICFEr nas últimas décadas, as taxas de reinternação permanecem muito altas.^25 , 26^ Os esforços para reduzir as readmissões e os gastos com saúde associados levaram os pesquisadores a investigarem os preditores de reinternação em pacientes com IC. Vários preditores de reinternação, incluindo pressões de enchimento elevadas, níveis aumentados de peptídeos natriuréticos e marcadores de ativação neuro-hormonal, foram descritos em pacientes com ICFEr até o momento.^27 - 29^ Por outro lado, um único modelo de predição de risco aplicável a todos os pacientes não foi estabelecido ainda.^24^ No presente estudo, descobrimos que o *strain* 3C apical e o uso de IECA foram os únicos preditores independentes de reinternação em pacientes com ICFEr avançada. Na verdade, o uso isolado do IECA não deve ser considerado motivo de internação. O uso de IECA na IC está entre os medicamentos que reduzem a mortalidade. Contudo, como estes pacientes se encontram em fases avançadas de IC, as funções renais deterioram-se ao longo do tempo, desenvolve-se a síndrome cardiorrenal e o processo torna-se um ciclo vicioso. A cada episódio de descompensação esse processo se agrava, levando ao aumento da frequência de internações e das taxas de mortalidade. O *strain* apical 3C apresentou sensibilidade moderada e baixa especificidade para predição de reinternação. Não detectamos associação entre reinternação e E/e’ SR e nível sérico de proBNP em nosso grupo de pacientes. Conforme afirmado por Desai SA e Stevenson LW em relatório especial, definir preditores de reinternação em pacientes com IC é um desafio, pois pode ser afetado por fatores psicossociais e socioeconômicos dos pacientes que podem ser facilmente ignorados nos estudos.^30 , 31^

### Limitações do estudo

O presente estudo tem algumas limitações. Primeiro, este é um estudo unicêntrico com uma população de pacientes relativamente pequena e acompanhamento de curto prazo. Em segundo lugar, a mortalidade por todas as causas foi utilizada como desfecho e as mortes por causas cardiovasculares não foram especificadas. Terceiro, tanto o proBNP sérico (424,00-85.524,00 ng/L) quanto o E/e’SR (46,92-1.966,67) tiveram uma faixa de distribuição muito ampla em toda a população do estudo, portanto, seus ORs foram próximos de 1,0 e os ICs foram muito estreitos. O parâmetro E/e’ SR é de difícil obtenção na prática clínica. Especialmente em pacientes que apresentam sintomas de descompensação durante a fase aguda, outros parâmetros de *strain* -eco semelhantes não puderam ser avaliados devido a fatores como escassez de equipamentos e recursos e difícil acesso. Essa situação pode ser considerada entre as limitações do estudo.

## Conclusão

Os resultados do presente estudo prospectivo demonstram que a E/e’ SR medida pelo STE é um preditor independente e sensível de mortalidade em curto prazo em pacientes com ICFEr avançada. Esses dados sugerem que a E/e’ SR pode ter um papel na identificação de pacientes com ICFEr em estágio terminal. Mais grandes estudos prospectivos são necessários para esclarecer esta associação.
