# Supplementary material for: Parâmetros de Strain Diastólico Estão Associados à Mortalidade em Curto Prazo e à Reinternação em Pacientes com Insuficiência Cardíaca Avançada
Source: Arq Bras Cardiol. 2024 Aug 14;121(8):e20230670. [Article in Portuguese] doi: 10.36660/abc.20230670 (PMC12092036; doi:10.36660/abc.20230670)
Supplement: Translation

# Translation: Diastolic Strain Parameters are Associated with Short Term Mortality and Rehospitalization in Patients with Advanced Heart Failure

**DOI:** 10.36660/abc.20230670i

**Authors:** Sefa Tatar, Abdullah İcli, Alpay Arıbaş, Nazire Belgin Akilli, Hakan Akilli, Ahmet Lütfi Sertdemir

**Affiliations:** 1 Turkey Necmettin Erbakan Universitesi – Kardiyoloji, Konya – Turkey; 2 Turkey Konya City Hospital, Konya – Turkey

**Keywords:** Heart Failure, Patient Readmission, Mortality

## Abstract

**Background:**

Heart failure (HF) is a leading cause of hospitalization and mortality worldwide and places a great economic burden on healthcare systems. Identification of prognostic factors in HF patients is of great importance to establish optimal management strategies and to avoid unnecessary invasive and costly procedures in end-stage patients.

**Objectives:**

In the current study, we aimed to investigate the association between diastolic strain parameters including E/e’ SR, and short-term outcomes in advanced HF patients.

**Methods:**

The population study included 116 advanced HF with reduced ejection fraction (HFrEF) patients. Clinical, laboratory, and echocardiographic evaluations of the patients were performed within the first 24 hours of hospital admission. Patients were followed for one month and any re-hospitalization due to worsening of HF symptoms and any mortality was recorded. The level of significance adopted in the statistical analysis was 5%.

**Results:**

E/e’ SR was significantly higher in the patient group compared to the control group (p=0.001). During one-month follow-up, 13.8% of patients died and 37.1% of patients were rehospitalized. Serum NT-ProBNP (p=0.034) and E/e’ SR (p=0.033) were found to be independent predictors of mortality and ACEİ use (p=0.027) and apical 3C strain (p=0.011) were found to be independent predictors of rehospitalization in the patient group.

**Conclusion:**

Findings of the current prospective study demonstrate that E/e’ SR measured by speckle tracking echocardiography is an independent and sensitive predictor of short-term mortality in advanced HFrEF patients and may have a role in the identification of end-stage HFrEF patients.

**Central Illustration:** : Diastolic Strain Parameters are Associated with Short Term Mortality and Rehospitalization in Patients with Advanced Heart Failure Diastolic strain echocardiography and short-term mortality in advanced HFrEF patients. HFrEF: heart failure with reduced ejection fraction; SR: strain rate; NT-ProBNP: The N-terminal prohormone of brain natriuretic peptide.

## Introduction

Heart failure (HF) is a major cause of fatal and non-fatal complications and continues to be a growing health problem all over the globe.^1^HF prevalence in the adult population is reported to be around 1% to 2% but the rate is over 10% in older individuals at and over 70 years of age.^2 , 3^Despite all new therapeutic options, HF is still associated with a high mortality rate and HF treatment places a great economic burden on healthcare systems.^4^Accurate assessment of clinical status and prognosis in HF patients is of paramount importance to establish appropriate management strategies and to avoid unnecessary invasive and costly procedures in end-stage patients.

Besides clinical evaluation and biochemical tests, echocardiographic evaluation is an indispensable part of HF assessment. Left ventricular systolic and diastolic functions are well-defined predictors for cardiovascular outcome in HF patients.^5 , 6^Speckle tracking echocardiography (STE) is a novel method for assessing left ventricular function via quantifying myocardial deformation (strain) and rate of deformation (strain rate).^7^The ratio of transmitral early filling velocity to early diastolic strain rate (E/e’ SR) measured by STE has also emerged as a reliable marker of left ventricular filling pressure and a sensitive predictor of cardiovascular outcomes in chronic HF patients.^8 , 9^On the other hand, there is limited data regarding the association between STE parameters including E/e’ SR and prognosis in advanced HF with severe symptoms (New York Heart Association Class III-IV functional status), severe cardiac dysfunction, and pulmonary or systemic congestion requiring intravenous diuretics.^10^

In the current study, we aimed to investigate the association between diastolic strain parameters including E/e’ SR measured by STE, and short-term outcomes in advanced HF patients.

## Methods

### Study population

In the current study, we enrolled 176 patients with advanced HF with reduced ejection fraction (HFrEF) (EF ≤40%, New York Heart Association Class III-IV functional status, pulmonary or systemic congestion requiring intravenous diuretics, more than two hospitalizations or recurrent emergency service visits in the past year, progressive deterioration in kidney function, development of cachexia without an identifiable cause, inability to use ACE inhibitors due to kidney failure or hypotension, worsening HF or intolerance to beta-blockers due to hypotension, development of resistance to diuretics, and increasing the furosemide dose above ≥160 mg/dL, as well as the development of hyponatremia) and 58 individuals without known cardiac disease.^10^There was no criterion defining the sample size used in the research, being stipulated for convenience. We did not have a criterion defining the sample size for each of the two groups examined. 45 advanced HFrEF patients were excluded from the study. Among the excluded patients, 10 had advanced aortic insufficiency, 5 had advanced mitral insufficiency, 3 had advanced aortic stenosis, and 7 had advanced mitral stenosis. Additionally, 10 patients were excluded from the study due to prosthetic aortic valve disease, and 5 due to prosthetic mitral valve disease. Furthermore, 5 patients were excluded from the study because they had an implantable cardioverter-defibrillator (ICD) installed and inotropic support requirement.

A total of 131 patients were referred to the echocardiographic evaluation. All patients underwent echocardiographic evaluation following their initial evaluation in the emergency room/outpatient clinic before admission to the intensive care unit (ICU)/ward and also before diuretic therapy. At this stage, another 15 patients were excluded from the study due to poor echocardiographic image quality. The final study population included 116 advanced HFrEF patients as the ‘patient group’ and 58 healthy individuals as the ‘control group’. Among 116 patients, 40 were admitted to the ICU and 76 were admitted to the cardiology ward following their initial evaluation. 3 of the patients who were admitted to the ward required transfer to the ICU during their hospitalization.

The flow chart of the study is presented in Figure 1 . N-terminal pro-brain natriuretic peptide (ProBNP) level was measured in all patients in addition to routine blood tests. Patients were followed for one month and any re-hospitalization due to worsening of HF symptoms and any mortality was recorded. Unfavorable outcomes of the patients were evaluated both by phone call and through medical records. The study protocol was approved by the local ethics committee (2019/1724) and informed consent was obtained from all patients.

**Figure 1:** – *Patient flow chart. 176 advanced HFrEF patients were enrolled in the study. 45 patients were excluded from the study due to the presence of severe heart valve disease, prosthetic heart valve, and pacemaker history. 15 patients were excluded due to poor echocardiographic image quality. The final study population included 116 advanced HFrEF patients.*

### Echocardiography

Transthoracic echocardiographic evaluation was performed using a Philips Epiq 7C ultrasound system (Bothell, WA, USA) with a 5-1 MHz transducer. Echocardiographic measurements were obtained by two experienced cardiologists using the standard techniques and images suggested by the American Echocardiography Association’s guidelines.^11^Left ventricular ejection fraction (LVEF) was calculated through Simpson’s biplane method. Parasternal long axis, parasternal short axis, apical 4 chamber, and apical long axis views were obtained. All images were larger than 60 fps and obtained for at least 5 cardiac cycles. Global longitudinal strain (GLS) values for all left ventricular walls were calculated. Both basal points and apex of the myocardium were determined on each window in SR images. The distance between the R peak of the QRS complex and the peak point of the mitral E rate and the distance between the R peak of the QRS complex and the peak point of the mitral A rate were calculated ( Figure 2 ). e’ SR values were calculated from the periods obtained ( Figure 3 ). Mitral inflow was measured at early diastolic wave (E) and late diastolic wave (A) at 5 to 10 cardiac cycles from 1 cm distal side of mitral valve ends on apical four-chamber view through pulsed wave (PW) Doppler, and their mean was calculated. The mitral E value was then divided into e’ SR rate, and the absolute value of E/e’ SR was calculated. If the variability of the SR measured by two operators was above 5%, the patient was excluded. If the difference between two measurements is below 5%, the arithmetic mean of two values was calculated and used for the analyses.

**Figure 2:** – *The relationship between strain velocity with the electrical activity of the heart on echocardiography. Electrical measurement times were taken from the peak point of the QRS wave to the peak points of the E wave and A wave on horizontal ground.*

**Figure 3:** – *E strain rate (1/s) and A strain rate (1/s) from the apical window.*

### Statistical analysis

SPSS (Statistical Package for Social Sciences) Windows 22.0 software was used for statistical analyses. The normal distribution of the continuous variables was assessed using Kolmogorov- Smirnov test. Data with normal distribution was expressed as mean± standard deviation, whereas data without normal distribution was expressed as median [interquartile range (IQR)]. Categorical variables were expressed as absolute (n) and relative frequencies (%) and the association between categorical variables was evaluated using the Chi-square test. The independent Student’s t-test was used to compare normally distributed parameters. Skewed parameters were compared using the Mann-Whitney U test. Logistic regression analyses were used to assess the prognostic value of GLS parameters for predicting re-hospitalization and mortality. Optimal cut-off values were determined by the analysis of the sensitivity and specificity values derived from the receiver operating characteristic (ROC) curve analysis. Any p-value below 0.05 (p<0.05) was accepted as statistically significant.

## Results

Demographic, clinical, echocardiographic, and laboratory characteristics of the patient and control groups are presented in Table 1 . Serum creatinine and urea levels were significantly higher in the patient group (p=0.018 and p=0.001, respectively). In addition, there were significant differences between the two groups regarding echocardiographic parameters including diastolic strain parameters ( Table 1 ). E/e’ SR was significantly higher in the patient group compared to the control group [188.8(323.0) vs 54.1(21.0), p=0.001].

**Table 1:** – Demographic, clinical, and laboratory characteristics of the patient and control groups

	Patient Group (n=116) Mean±SD/Median (IQR)	Control Group (n=58) Mean±SD/Median (IQR)	p
**Parameters**			
**Age (years)**	67.7±12.8	68.1±11.2	0.810
**Gender**			0.524
Male, n (%)	71 (61.2%)	36 (62.1%)	
Female, n (%)	45 (38.8%)	22 (37.9%)	
**CAD, n (%)**			**0.019**
None	41 (35.3%)	35 (60.3%)	
Medical	15 (12.9%)	5 (8.6%)	
PTCA	39 (33.6%)	11 (19%)	
CABG	21 (18.1%)	7 (12.1%)	
**Hypertension, n (%)**	84 (72.4%)	47 (81%)	0.145
**Smoking, n (%)**	40 (34.5%)	15 (25.9%)	0.321
**Diabetes mellitus, n (%)**	52 (44.8%)	34 (58.6%)	0.06
**Hyperlipidemia, n (%)**	49 (42.2%)	23 (39.7%)	0.43
**Stroke, n (%)**	16 (13.8%)	6 (10.3%)	0.35
**CKD, n (%)**	53 (45.7%)	21 (36.2%)	0.151
**Hemoglobin (g/dl)**	12.4±2.2	12.9±2.2	0.828
**Hematocrit**	38.4±6.7	39.8±6.4	0.364
**MCV (fL)**	83.7±7.5	86.5±6.6	0.275
**Platelet Count (x109/L)**	246.5±96.3	252.0±78.3	0.705
**Sodium (mmol/L)**	138.0±3.7	138.8±3.4	0.837
**Potassium (mmol/L)**	4.6±0.7	4.3±0.6	0.182
**GFR (ml/min)**	51(29)	72(40)	**0.002**
**Urea (mg/dl)**	57(39)	40(29)	**0.001**
**Creatine (mg/dl)**	1.39(1)	0.98(1)	**0.018**
**AST (U/L)**	18(12)	17(13)	0.870
**ALT (U/L)**	15(14)	17(18)	0.908
**WBC Count (x109/L)**	9.1 (3.9)	8.3(4.5)	0.308
**Echocardiography**			
**End diastolic diameter (mm)**	56.8±7.7	45.3±4.5	**0.001**
**End systolic diameter (mm)**	44.3±8.7	27.8±4.0	**0.001**
**Left atrium (mm)**	43.5±7.0	34.8±6.4	**0.001**
**Pulmonary arterial pressur (mmHg)**	35(21)	30(9)	**0.001**
**End diastolic volume (ml)**	182.8(90)	42.4(32)	**0.001**
**End systolic volume (ml)**	122.5(60)	17.3(14)	**0.001**
**Ejection fraction (%)**	30.6(11)	57.1(13)	**0.001**
***Apical 3C strain (%)***	-6.7±2.9	-9.3 ±3.2	**0.001**
***Global longitudinal strain (%)***	-7.2±2.46	-10.5±2.6	**0.001**
**e SR, 1/s**	0.34(0.4)	1.0(0.5)	**0.001**
**a SR, 1/s**	0.3(0)	1.1(1)	**0.001**
**E/e’ SR**	188.8(323.0)	54.1(21.0)	**0.001**

*ALT: alanine aminotransferase; AST: aspartate aminotransferase; CABG: coronary artery bypass grafting; CAD: coronary artery disease; CKD: chronic kidney disease; GFR: glomerular filtration rate; MCV: mean corpuscular volume; PTCA: percutaneous transluminal coronary angioplasty; SR: strain rate; WBC: white blood cell. Bold values indicate significant p values.*

### Predictors of mortality in the patient group

During one month follow-up 16 (13.8%) patients died. Demographic, clinical, echocardiographic, and laboratory characteristics of the mortality and survival groups are presented in Table 2 . Beta-blocker and angiotensin-converting enzyme inhibitor (ACEi) use was less common in the mortality group. On the other hand, serum proBNP level was significantly higher in the mortality group. In addition, E/e’ SR was also significantly higher in the patient group compared to the control group. Left ventricular end-diastolic and left atrial diameters were found to be significantly lower in the mortality group

**Table 2:** – Demographic, laboratory and echocardiographic characteristics of the mortality and survival groups

	Mortality Group (n=16) Mean±SD/Median (IQR)	Survival Group (n=100) Mean±SD/Median (IQR)	p
**Age, (years)**	69.1±12.4	67.5±12.9	0.644
**Gender**			0.236
Male, n (%)	8 (50.0%)	63 (63.0%)	
Female, n (%)	8 (50.0%)	37 (37.0%)	
**Total length of hospital stay (days)**	7 (4-9)	7 (3-9)	0.690
**Hypertension, n (%)**	10 (62.5%)	74 (74%)	0.251
**Diabetes mellitus, n (%)**	7 (43.8%)	45 (45%)	0.573
**Hyperlipidemia, n (%)**	5 (31.2%)	44 (44%)	0.249
**Beta blocker, n (%)**	8 (50%)	75 (75%)	**0.043**
**Diuretic, n (%)**	8 (50%)	69 (69%)	0.115
**ACEi, n (%)**	5 (31.2%)	59 (59%)	**0.036**
**MRA, n (%)**	3 (18.8%)	34 (34%)	0.178
**End diastolic diameter (mm)**	52.7±8.7	57.4±7.3	**0.021**
**End systolic diameter (mm)**	42.6±9.1	44.6±8.6	0.384
**Left atrium diameter (mm)**	40.4±8.2	44.0±6.6	**0.049**
**GFR (ml/min)**	41 (34)	51 (20)	0.216
**ProBNP (ng/L)**	13800 (30747)	4860 (9174)	**0.002**
**End diastolic volume (ml)**	166.6 (138)	182.3 (76)	0.876
**End systolic volume (ml)**	116.4 (97)	121.2 (51)	0.719
**Ejection fraction (%)**	31.7 (10)	30.3 (11)	0.513
**Apical 4C strain (%)**	-7.5±2.4	-7.3±2.7	0.777
**Apical 3C strain (%)**	-6.2±2.3	-6.7 ±3.0	0.623
**Apical 2C strain (%)**	-7.4±4.2	-6.7±2.8	0.503
**Global longitudinal strain (%)**	-7.4±2.1	-7.2±2.5	0.742
**E SR, 1/s**	0.1 (0.3)	0.36 (0.3)	**0.004**
**A SR, 1/s**	0.3 (0.0)	0.3 (0.0)	0.774
**E/e’ SR**	600.0 (679.0)	184.4 (318.0)	**0.009**

*ACEi: Angiotensin converting enzyme inhibitor; BNP: brain natriuretic peptide: GFR: glomerular filtration rate; MRA: mineralocorticoid receptor antagonist; SR: strain rate. Bold values indicate significant p values.*

Univariate and multivariate logistic regression analyses were performed for identifying predictors of mortality in the patient group and the results of these analyses are given in Table 3 . Multivariate regression analysis revealed that serum proBNP level and E/e’ SR were independent predictors of mortality in the patient group during one-month follow-up.

**Table 3:** – Univariate and multivariate regression analyses for identifying predictors of mortality in the patient group

Parameters	Univariate analysis		p	Multivariate analysis		p
	OR	95% CI		OR	95% CI	
Beta blocker*, n (%)	3.0	1.019-8.829	**0.046**	2.753	0.824-9.203	0.100
Diuretic*, n (%)	2.226	0.765-6.474	0.142	0.861	0.157-4.714	0.863
ACEi*, n (%)	3.166	1.023-9.798	**0.046**	1.356	0.306-5.998	0.688
**MRA, n (%)**	2.232	0.595-8.372	0.234	-	-	-
End diastolic diameter* (mm)	0.916	0.850-0.988	**0.024**	0.956	0.866-1.055	0.369
**End systolic diameter (mm)**	0.972	0.913-1.036	0.381	-	-	-
**LVEF (%)**	0.983	0.927-1.043	0.569	-	-	-
Left atrium diameter* (mm)	0.927	0.858-1.001	**0.053**	0.925	0.851-1.004	0.064
ProBNP* (ng/L)	1.000	1.000-1.000	**0.029**	1.000	1.000-1.000	**0.034**
E/e’ SR*	1.001	1.000-1.001	**0.048**	1.001	1.000-1.001	**0.033**

ACEi: angiotensin converting enzyme inhibitor; BNP: brain natriuretic peptide; CI: confidence interval; LVEF: left ventricular ejection fraction; MRA: mineralocorticoid receptor antagonist; OR: odds ratio; SR: strain rate. * This parameters are inculeded in the multivariate analysis. Bold values indicate significant p values.

Receiver operating characteristic analysis demonstrated that a cut-off value of 218.75 for E/e’ SR had a sensitivity and specificity of 86.7 and 58.0% for predicting mortality in the patient group. A cutoff value of 6326.50 ng/L for serum proBNP level had a sensitivity and specificity of 85.7 and 56.2% for predicting mortality.

### Predictors of rehospitalization in the patient group

During one month follow-up 43 (37.1%) patients were rehospitalized due to worsening HF symptoms. Demographic, clinical, echocardiographic, and laboratory characteristics of the rehospitalization and non-rehospitalization groups are shown in Table 4 . ACEi use was more common in the rehospitalization group. In addition, apical 3C strain was significantly worse in the hospitalization group compared to the non-rehospitalization group. On the other hand, serum proBNP levels and E/e’ SR were found to be similar between the groups.

**Table 4:** – Demographic, laboratory, and echocardiographic characteristics of the rehospitalization and non-rehospitalization groups

	Rehospitalization Group (n=43) Mean±SD/Median (IQR)	Non-rehospitalization Group (n=73) Mean±SD/Median (IQR)	p
**Age, (years)**	66.5±11.9	68.4±13.3	0.452
**Gender**			0.845
Male, n (%)	27 (62.8%)	44 (60.3%)	
Female, n (%)	16 (37.2%)	29 (39.7%)	
**Total length of hospital stay (days)**	6 (3-9)	7 (3-9)	0.662
**Hypertension, n (%)**	34 (79.1%)	50 (68.5%)	0.155
**Diabetes mellitus, n (%)**	24 (55.8%)	28 (38.4%)	0.551
**Hyperlipidemia, n (%)**	19 (44.2%)	30 (41.1%)	0.447
**Beta blocker, n (%)**	33 (76.7%)	50 (68.5%)	0.231
**Diuretic, n (%)**	32 (74.4%)	45 (61.6%)	0.114
**ACEi, n (%)**	29 (67.4%)	35 (47.9%)	**0.032**
**MRA, n (%)**	17 (39.5%)	20 (27.4%)	0.126
**End diastolic diameter (mm)**	57.1±7.1	56.5±8.0	0.686
**End systolic diameter (mm)**	44.1±8.7	44.5±8.7	0.824
**Left atrium diameter (mm)**	44.4±7.1	43.0±6.6	0.292
**GFR (ml/min)**	47.0 (17.0)	51.5 (34.0)	0.224
**ProBNP (ng/L)**	5560 (8721)	7368 (15593)	0.554
**End diastolic volume (ml)**	196.8 (92)	169.8 (78)	0.154
**End systolic volume (ml)**	129.9 (64)	117 (48)	0.117
**Ejection fraction (%)**	33 (11)	30 (11)	0.268
***Apical 4C strain (%)***	-6.7±2.4	-7.63±2.7	0.116
***Apical 3C strain (%)***	-5.57±2.7	-6.7 ±3.0	**0.010**
***Apical 2C strain (%)***	-6.4±2.9	-6.9±2.9	0.457
***Global longitudinal strain (%)***	-7.0±2.6	-7.4±2.36	0.462
**E SR, 1/s**	0.37 (0.3)	0.32 (0.4)	0.817
**A SR, 1/s**	0.32 (0)	0.3 (0)	0.765
**E/e’ SR**	178.3 (305)	221.8 (473)	0.470

*ACEi: angiotensin-converting enzyme inhibitor; BNP: brain natriuretic peptide; GFR: glomerular filtration rate; MRA: mineralocorticoid receptor antagonist; SR: strain rate. Bold values indicate significant p values.*

Univariate and multivariate regression analyses were performed for identifying predictors of rehospitalization in the patient group and the results of these analyses are given in Table 5 . Multivariate regression analysis revealed that ACEi use and apical 3C strain were independent predictors of rehospitalization in the patient group during one-month follow-up.

**Table 5:** – Univariate and multivariate regression analyses for identifying predictors of rehospitalization in the patient group

Parameters	Análise univariada		p	Análise multivariada		p
	OR	95% CI		OR	95% CI	
**Hipertensão, n (%)**	0.575	0.237-1.395	0.221	-	-	-
Diurético*, n (%)	0.552	0.240-1.269	0.162	0.756	0.269-2.218	0.597
ECAİ*, n (%)	0.445	0.203-0.976	**0.043**	2.525	1.111-5.738	**0.027**
ARM*, n (%)	0.577	0.260-1.283	0.177	0.832	0.317-2.186	0.709
**Volume diastólico final (ml)**	1.003	0.997-1.009	0.264	-	-	-
**Volume sistólico final (ml)**	1.005	0.997-1.012	0.247	-	-	-
**FEVE (%)**	0.969	0.923-1.016	0.192	-	-	-
Strain apical 3C* (%)	1.138	1.023-1.265	**0.017**	1.154	1.033-1.288	**0.011**
Strain apical 4C* (%)	1.119	1.008-1.241	**0.035**	1.039	0.898-1.202	0.609

IACEi: angiotensin-converting enzyme inhibitor; CI: confidence interval; LVEF: left ventricular ejection fraction; MRA: mineralocorticoid receptor antagonist; OR: odds ratio. *These parameters are included in the multivariate analysis. Bold values indicate significant p values.

Receiver operating characteristic analysis demonstrated that a cut-off value of -5.55% for apical 3C strain had a sensitivity and specificity of 76.7 and 50.7% for predicting rehospitalization in the patient group. The Central Illustration summarizes the main information of the manuscript.

## Discussion

In the current prospective study, we revealed that E/e’ SR measured by STE and serum proBNP level were independent predictors of mortality in advanced HFrEF patients during one-month follow-up. On the other hand, apical 3C strain was the only predictor of rehospitalization in the same patient group. There are other studies in the literature investigating the prognostic role of STE parameters including E/e’ SR in HF patients but our study is unique for its patient population (advanced HFrEF patients) and timing of echocardiographic evaluation (within the first 24 hours following hospital admission).^9^

In HF patients, myocardial relaxation is impaired, and left ventricular filling decreases. As a result, there is an increase in diastolic pressure and patients begin to develop symptoms of HF. With increased venous tone, development of sodium retention, and activation of neuro-hormonal pathways, left ventricular diastolic pressure increases further, left ventricular stiffness increases significantly, and pulmonary edema occurs in patients. Although the atrial pressure contribution tries to increase cardiac output to compensate for this situation, diastolic filling is limited due to a harsh ventricular response. This situation causes exertion dyspnea and pulmonary congestion to worsen in patients. Especially left atrial dilatation and dysfunction should be noted as an important guide in patients with diastolic HF.^12 , 13^Although pathophysiological explanations have been tried to be explained in this way, the pathophysiology has still not been clearly explained.

Myocardial deformation imaging with STE provides important additional diagnostic and prognostic information over basic echocardiography and tissue Doppler imaging in HF patients.^14^Previous studies have shown that GLS was an independent predictor of all-cause mortality in the HFrEF patients and added significant incremental prognostic value to the well-known risk factors such as LVEF.^15 , 16^On the other hand, in the present study, we did not detect an association between GLS and mortality/rehospitalization in advanced HFrEF patients. The most important difference between these studies and ours is that we investigated a sample of advanced HFrEF patients but other studies had a global HF population. This discrepancy may also be a result of the relatively small patient population of the current study.

In their large series evaluating clinical characteristics and outcomes of patients with advanced HF, Javaloyes et al. reported that most of the patients had a ‘warm and wet’ phenotype.^17^The 1-year mortality rate was 30.8% in their study group, and highest in the patients with a ‘cold and dry’ phenotype. As a result, they concluded that hypoperfusion was related to an increased in-hospital and 1-year mortality rate in advanced HF patients, in line with the previous studies.^18^On the other hand, in patients without hypoperfusion, predictors of mortality are not clear. In the current study, we revealed that early echocardiographic evaluation and non-invasive assessment of left ventricular filling pressure via the ratio of transmitral early filling velocity to early diastolic strain rate (E/e’ SR), in addition to clinical and biochemical parameters, may provide valuable insight about the prognosis of advanced HF patients with ‘warm and wet’ phenotype.

The ratio of transmitral early filling velocity to early diastolic strain rate (E/e’ SR) obtained by STE has emerged as a reliable measure of left ventricular filling pressures that circumvents technical limitations of the Doppler-derived parameters.^19 , 20^E/e’ SR is a less load-dependent parameter than E/e’ and is not affected significantly by volume overloading which makes it a useful marker of left ventricular filling pressure in HFrEF patients. Recent studies have shown that E/e’ SR was a strong predictor of mortality and worse outcomes in various conditions including HFrEF, atrial fibrillation, aortic stenosis, and type 2 diabetes.^9 , 21 - 24^In line with these findings, we revealed that E/e’ SR was an independent predictor of short-term mortality in advanced HFrEF patients together with serum proBNP level. Both E/e’ SR and serum proBNP levels had a high sensitivity but a low specificity for the prediction of mortality in our patient group.

Despite tremendous improvements in the management strategies of HFrEF patients in the last decades, rehospitalization rates remain very high.^25 , 26^Efforts to reduce readmissions and associated healthcare expenditures have prompted researchers to investigate the predictors of rehospitalization in HF patients. Various predictors of rehospitalization including elevated filling pressures, increased levels of natriuretic peptides, and markers of neurohormonal activation have been described in HFrEF patients to date.^27 - 29^On the other hand, a single risk prediction model applicable to all patients has not been established yet.^24^In the current study, we found that apical 3C strain and ACEi use were the only independent predictors of rehospitalization in advanced HFrEF patients. Actually, the use of ACEi alone should not be considered as a reason for hospitalization. The use of ACEi in HF is among the drugs that reduce mortality. However, since these patients are in advanced stages of HF, renal functions deteriorate over time, cardiorenal syndrome develops, and the process becomes a vicious cycle. With each decompensation episode, this process worsens, leading to increased hospitalization frequency and mortality rates. Apical 3C strain had a moderate sensitivity and a low specificity for prediction of rehospitalization. We did not detect an association between rehospitalization and E/e’ SR and serum proBNP level in our patient group. As stated by Desai SA and Stevenson LW in a special report, defining predictors of rehospitalization in HF patients is challenging in that it can be affected by psychosocial and socioeconomic factors of the patients that can be easily overlooked in the studies.^30 , 31^

### Study limitations

The current study has some limitations. First, this is a single-center study with a relatively small patient population and short-term follow-up. Second, all-cause mortality was used as an end point and deaths due to cardiovascular causes were not specified. Third, both serum proBNP (424.00-85524.00 ng/L) and E/e’SR (46.92-1966.67) had a very wide distribution range throughout the study population, thus their ORs were close to 1.0 and CIs were very narrow. The parameter E/e’ SR is difficult to obtain in clinical practice. Especially in patients presenting with decompensation symptoms during the acute phase, other similar strain echo parameters could not be evaluated due to factors such as equipment and resource shortages, and difficult access. This situation can be considered among the limitations of the study.

## Conclusion

Findings of the current prospective study demonstrate that E/e’ SR measured by STE is an independent and sensitive predictor of short-term mortality in advanced HFrEF patients. This data suggests that E/e’ SR may have a role in the identification of end-stage HFrEF patients. Further large prospective studies are required to clarify this association.
